# Deconvolution of Complex 1D NMR Spectra Using Objective Model Selection

**DOI:** 10.1371/journal.pone.0134474

**Published:** 2015-08-04

**Authors:** Travis S. Hughes, Henry D. Wilson, Ian Mitchelle S. de Vera, Douglas J. Kojetin

**Affiliations:** 1 Department of Molecular Therapeutics, The Scripps Research Institute, Scripps Florida, Jupiter, Florida, 33458, United States of America; 2 Graduate Program, The Scripps Research Institute, Scripps Florida, Jupiter, Florida, 33458, United States of America; The Francis Crick Institute, UNITED KINGDOM

## Abstract

Fluorine (^19^F) NMR has emerged as a useful tool for characterization of slow dynamics in ^19^F-labeled proteins. One-dimensional (1D) ^19^F NMR spectra of proteins can be broad, irregular and complex, due to exchange of probe nuclei between distinct electrostatic environments; and therefore cannot be deconvoluted and analyzed in an objective way using currently available software. We have developed a Python-based deconvolution program, *decon1d*, which uses Bayesian information criteria (BIC) to objectively determine which model (number of peaks) would most likely produce the experimentally obtained data. The method also allows for fitting of intermediate exchange spectra, which is not supported by current software in the absence of a specific kinetic model. In current methods, determination of the deconvolution model best supported by the data is done manually through comparison of residual error values, which can be time consuming and requires model selection by the user. In contrast, the BIC method used by *decond1d* provides a quantitative method for model comparison that penalizes for model complexity helping to prevent over-fitting of the data and allows identification of the most parsimonious model. The *decon1d* program is freely available as a downloadable Python script at the project website (https://github.com/hughests/decon1d/).

## Introduction

Early NMR studies of biomacromolecules were performed on relatively simple low molecular weight model systems using one-dimensional (1D) methods. However, in the past 30 years, significant advances in isotope labeling, NMR pulse sequence development and software methods paved the path for multidimensional NMR studies of biomacromolecules. Despite the significant advances made using multidimensional NMR studies, there has been a recent resurgence in 1D NMR methods, in particular fluorine (^19^F) NMR, to study complex biomolecular interactions in particular because this method can simplify NMR spectra to one or a few NMR detectable nuclei [1–3].

To facilitate ^19^F protein NMR studies, a fluorine probe is attached to a unique site, or several sites, on the protein either via a biosynthetic route (e.g. fluorotryptophan) or through the use of a cysteine-conjugated fluorine tag [2]. Although this type of labeling can theoretically produce a single 1D ^19^F NMR signal, e.g. if only one fluorine tag is attached, it might also yield complex spectra due to various phenomena, including irreversible molecular inhomogeneity (e.g. posttranslational protein modification or protein degradation) or reversible processes such as slow conformation exchange between multiple conformations or ligand-associated states. Although irreversible molecular inhomogeneity must be considered, here we focus on methods for interpreting 1D NMR spectra for systems in exchange between multiple conformational states.

Biomolecules, such as proteins, switch between different conformations, and in general the atoms that make up the protein switch between different chemical shift environments. The difference in chemical shift between the environments and the rate of exchange between them determines the appearance of the NMR signal. This can result in a single sharp peak when the rate of exchange is fast (ps-ns exchange), a broadened peak when the rate of exchange is comparable to the chemical shift (in frequency units), or can appear as multiple and possibly overlapping peaks at unique chemical shift values when exchange is slow compared to the chemical shift difference between environments [4]. Deconvolution of intermediate to slow exchange data, where complex 1D NMR signals are observed, is desirable because it can give information about the number and fractional occupancy of the distinct states. The chemical shift separation of fitted peaks also provides an upper bound estimate for the exchange rate between conformations. Thus the ^19^F label can help define the regional dynamics over a broad timescale.

Several recent studies have used small, cysteine-conjugating fluorine probes, such as 2,2,2 trifluoroethanethiol (TET) or 3-bromo-1,1,1-trifluoroacetone (BTFA) to perform ^19^F NMR studies to characterize the conformational ensemble of proteins. This includes important work on rhodopsin [5], diacyl glycerol kinase (DAGK) [6], as well as the interaction of ligands with the β2-adrenergic receptor (β2AR) [7, 8]. The β2AR studies found evidence consistent with slow conformational exchange of single fluorine probes between two or more structural states. In these studies, deconvolution of the fluorine signal was performed using commercially available software designed primarily to analyze high-resolution small molecule 1D NMR data. This type of analysis typically requires the user to determine the initial location and number of peaks that make up the underlying signal. With this input, a fit is then made and a measure of goodness of fit, commonly residual error, is computed. Addition of peaks will usually reduce the residual error to the point of overfitting, where noise is fit instead of the underlying signal. Thus the user is often forced to determine when the fit is “good enough” and choose the number of peaks in the final fit. This user-determined model selection can be time consuming and require other experiments to confirm the deconvolution. Thus, an objective method of model selection would be beneficial.

Here, we present a method that utilizes residual error minimization in concert with a Bayesian information criterion (BIC) [9] score to automate peak placement, identify the number of signals, and determine peak line widths for 1D NMR spectra in the absence and presence of chemical exchange. BIC can be used to determine the most parsimonious model out of a set of calculated models. Differences in BIC values can indicate the degree to which one model is favored over another [9, 10]. Here, we test the robustness of this approach on simulated 1D NMR spectra. In addition we use this method to analyze experimental 1D ^19^F NMR spectra from peroxisome proliferator-activated receptor gamma (PPARγ), where the ligand-free form of this protein contains multiple overlapping NMR resonances.

## Results and discussion

### Spectral deconvolution program description

**Fig 1:**
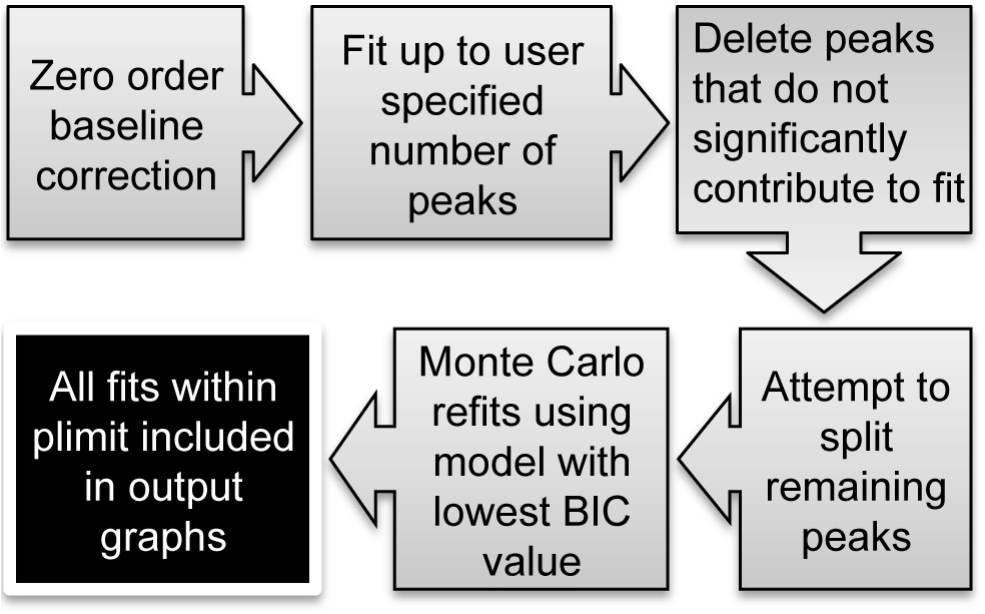
Schematic outline of the fitting protocol adopted by *decon1d*.

We developed a program, *decon1d*, written in the Python programming language with the main purpose of presenting a lower bound for the number of distinct spectral peaks or chemical shift environments that make up the overall NMR spectrum. The input file format should contain the left and right limits of the spectrum (in p.p.m.), the number of data points, and the intensity values (y-axis values) of each point in the spectrum to be analyzed. An example of the input format is displayed in S1 Fig (see Methods for details). An overview of the fitting method in *decond1d* is shown in Fig 1.

First, the input data baseline is adjusted (detailed in Methods) and then the initial fit is accomplished through progressive peak placement at the areas of greatest number of consecutive positive residual values and/or at the point of the highest positive residual value; residual value is the difference between the data value and the model/fitted value. Minimization is performed using the *lmfit* implementation of the Levenburg-Marquadt least squares fitting procedure (http://lmfit.github.io/lmfit-py/), which allows user defined bounds on any fitted parameter (i.e. peak width, height, chemical shift and phase). Next, a Bayesian information criterion (BIC) score [9] is calculated for the fit. This is repeated up to a model consisting of the user-specified maximum number of peaks—in our experience, it is best to set this to a value more than the anticipated number of peaks to assure that all non-noise peaks are fit; we recommend about twice the number of apparent peaks. As more peaks are fit, the residual error in general decreases. In contrast, the unitless BIC score, which incorporates penalties for each additional peak, decreases as the new peaks significantly improve the fit—until the point at which the fit is not improved, and consequently the BIC score increases as new peaks are incorporated. The model with the most peaks that has a lower BIC score than a model with one fewer peak is then chosen for further refinement. Each peak within this initial model is deleted one at a time, a new minimization is performed, and the BIC value is calculated for the new model. Peaks are progressively deleted until the BIC value of the new model, with one fewer peak, is larger by more than a specified limit (defined as “*plimit*”; default = 15) when compared to the current best BIC value. This is a rather strict default value, which should produce a model with a minimal number of peaks needed to explain the data. To put *plimit* = 15 in perspective, it has been suggested that a difference in BIC value lower than 2 provides little evidence that a given model is favored over another while a difference higher than 6 constitutes “strong” (20:1 posterior odds) and a difference higher than 10 constitutes “very strong” (150:1 posterior odds) evidence that a given model is favored over another [10]. A BIC difference of 15 constitutes evidence that the model is favored over another by ~1800:1 [9]. BIC values can only be meaningfully compared between models that are fit to the exact same data set.

After the above procedure, further improvements to the current best model are attempted via splitting of component peaks. That is, a randomly selected single peak from the current model is split into two peaks with chemical shifts and summed area similar to the parent peak and the whole model is re-minimized. If this new model, with one additional peak, is the same or better than the previous model as judged by the BIC value, then the additional peak is kept; otherwise it is discarded and the original peak is reinstated. This splitting is continued until the new BIC value increases by more than two as compared to the best BIC value. Monte Carlo analysis is then carried out on the model with the overall lowest BIC value; the number of Monte Carlo refits attempted can be user-specified. This procedure involves taking the model and perturbing all of the composite peak parameters at once and refitting (see Methods). All models from any part of this procedure with a BIC value less than *plimit* more than the final best scoring model are then presented to the user with the corresponding BIC value (by default as matplotlib-based graphs and/or exported as PDF/image files). Models that differ in BIC value by less than 2 should be considered equally good models. It should be noted that in general the best and second best model, as judged by BIC value, are well separated. All of the example *decon1d* fits presented here have a BIC value at least 4 less than the next best model, and in only a few cases did the best and second best model differ in the BIC value by less 6; these cases are indicated. In contrast the Monte Carlo refits of the best model, in which the number of peaks stays the same, tend to be similar and group closely around the best model. For this reason the Monte Carlo results are output from *decon1d* overlaid on a single separate graph. In the case where a Monte Carlo fit results in a decrease in the BIC value of more than 2, these fits are graphed separately. In all the fits presented here the Monte Carlo analysis resulted in very similar models (BIC difference less than 2), which in the majority of cases are indistinguishable by eye. In the interest of clarity only the lowest BIC model from this group of Monte Carlo results is presented in this report. Separation of the fitting and graphing parts of the program is anticipated in a future version.

### Initial test of fitting procedure

**Fig 2:**
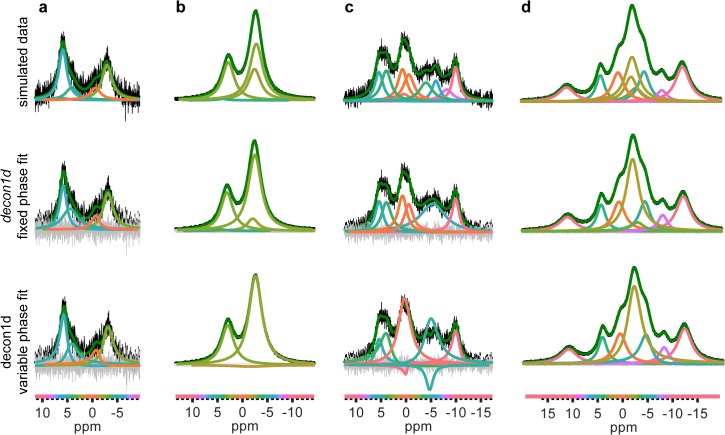
Fitting of simulated spectra with *decon1d*. a-d) Non-exchange broadened spectra of varying signal-to-noise ratio and number, width, frequency and height of component peaks were simulated (top row). *decon1d* was then used to fit these simulated spectra allowing for either fixed phase (middle row) or variable phase (bottom row). The color of the component peaks identified in each fit serves as a visual aid for comparisons between fits as it identifies the approximate chemical shift of the peak center, indicated by the colored bar on the bottom. The difference between the data and the fit (residual error) is shown in grey and the sum of individual fitted peaks is shown in green. An alternate deconvolution of the variable phase fit for column c is displayed in S5 Fig.

To test the utility and robustness of the *decon1d* program, we generated simulated Lorentzian peak NMR spectra using MATLAB with randomly chosen number of peaks (generated from a uniform distribution in the interval [0 12]), width (generated from a normal distribution where μ = 0.5 ppm, σ = 5 ppm), center (generated from a normal distribution where μ = 0 ppm, σ = 5 ppm), and intensity (generated from a normal distribution where μ = 1, σ = 0.6, then normalized). These simulated spectra were fit using *decon1d* with the phase of each peak limited to a range within ± π/50 radians. The results, which are a series of spectral predictions, were compared to the starting simulated spectra. In all cases the best model contained fewer or the same number of peaks that comprised the simulated spectrum (Fig 2 and S2–S4 Figs). In cases where an isolated, well-resolved peak can be detected by eye, the fit is excellent (e.g. Fig 2A and 2B). In cases where peaks are overlapping, the program consistently underestimates the number of peaks that a given spectral area comprises, combining several underlying peaks into one fitted peak (e.g. Fig 2C and 2D); thus the *decon1d* fitting procedure provides a lower bound on the true number of peaks. In *decon1d*, limiting the phase of the component peaks in each model to ≤ ± π/50 radians is considered for statistical purposes as ‘fixed phase’. Importantly, allowing the phase to vary over a wider interval is necessary for fitting intermediate exchange and mis-phased data (*vide infra*).

We also performed deconvolution of these simulated spectra using the commercially available program iNMR, which is the only deconvolution program that does not require imputation of a user-defined kinetic scheme for exchange and that allows ASCII file input, which is the format of our simulated data. In this comparison we did not specify peak locations in deconvolutions utilizing iNMR, but used the automatically placed peaks in order to minimize user bias in the output, similar to *decon1d*. In general iNMR, when used in this way, did not provide a lower bound on the number of peaks and at times overfit dramatically (S2–S4 Figs).

Chemical exchange effects were not modeled in the simulated spectra of Fig 2. We therefore tested the effect of loosening the phase limits of component peaks beyond ± π/50 radians when fitting data without chemical exchange effects. Most models that allowed the phase to vary beyond ± π/50 radians were similar to fits using the fixed phase method, with only a few models that were substantially different (Fig 2 and S3 and S4 Figs). In all the cases where variable phase resulted in a less accurate fit the problematic regions contained multiple overlapping peaks. Thus allowing variable phase may result in less accurate fits of some complex spectra, but may be necessary if intermediate exchange effects are likely (discussed further below). However, when we compared the BIC values for the best variable phase fits vs. fixed phase fits, in all cases the fixed phase fit BIC value was at least 5 lower than the variable phase model BIC value. This indicates that these non-exchange broadened correctly phased simulated spectra are better fit in these simulations by a fixed phase model, as expected. Therefore, in practical use it may be necessary to generate two models for each spectrum, and then compare variable phase vs. fixed phase BIC values to determine the best overall model. It should be noted that when comparing BIC values a difference less than 2 does not support one model over another. Rather a larger difference lends more support for one model versus another [10].

**Fig 3:**
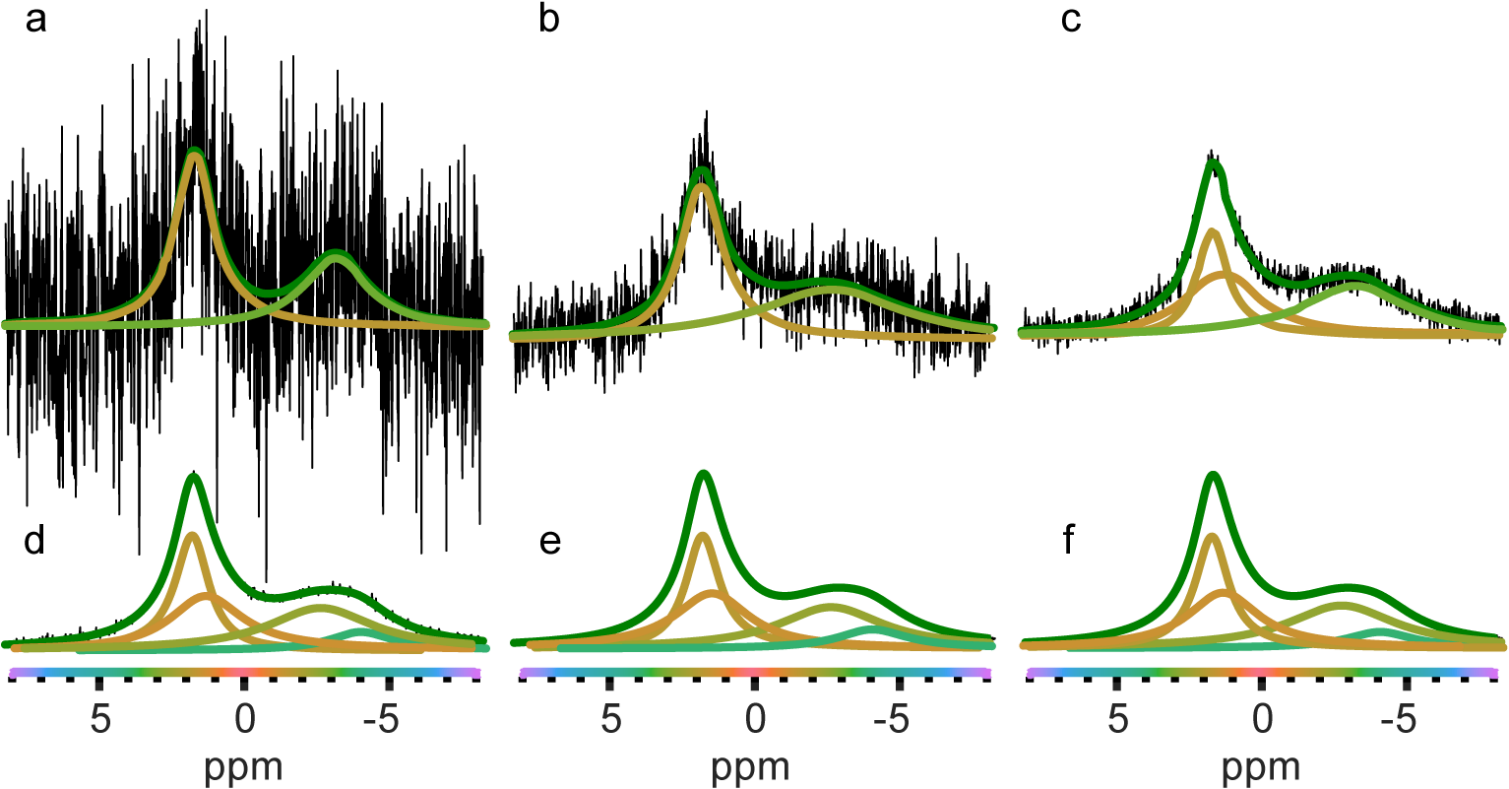
Lower signal-to-noise ratio leads to decreased peak assignment. a-e) Fits of simulated data with signal-to-noise ratio: a) 5 b) 10, c) 25, d) 75 and e) 244. f) Input simulated NMR spectra showing the true underlying peaks that make up the spectra. Signal-to-noise was calculated from the highest signal value divided by the root mean square value of the noise in a region devoid of signal. An alternate fit of the lowest signal to noise data (panel a) was found with a BIC value 4.67 higher than the model shown, with the only substantial difference being that the prediction of rightmost peak chemical shift is -3.58 ppm (not shown) rather than -3.12 ppm (shown).

We also tested the ability of the method to deconvolute simulated spectra with varying signal-to-noise ratio. As the signal-to-noise ratio decreases, the number of peaks fit to a given spectrum either decreases or stays the same (Fig 3). Again, fixed phase fits had lower BIC values than variable phase fits (not shown), indicating correctly that the simulated spectrum does exhibit intermediate exchange effects and is correctly phased. It should be noted that the digital resolution of the spectrum should be much higher (i.e the Gaussian noise fluctuations are much narrower) than the minimum peak width limit (set by the user) in order to avoid fitting of the noise. Deconvolution using iNMR yielded less consistent results, with the number of fit peaks varying markedly for the same simulated spectra at differing signal to noise ratios (S6 Fig).

### Impact of incorrect phasing on the fitting procedure

**Fig 4:**
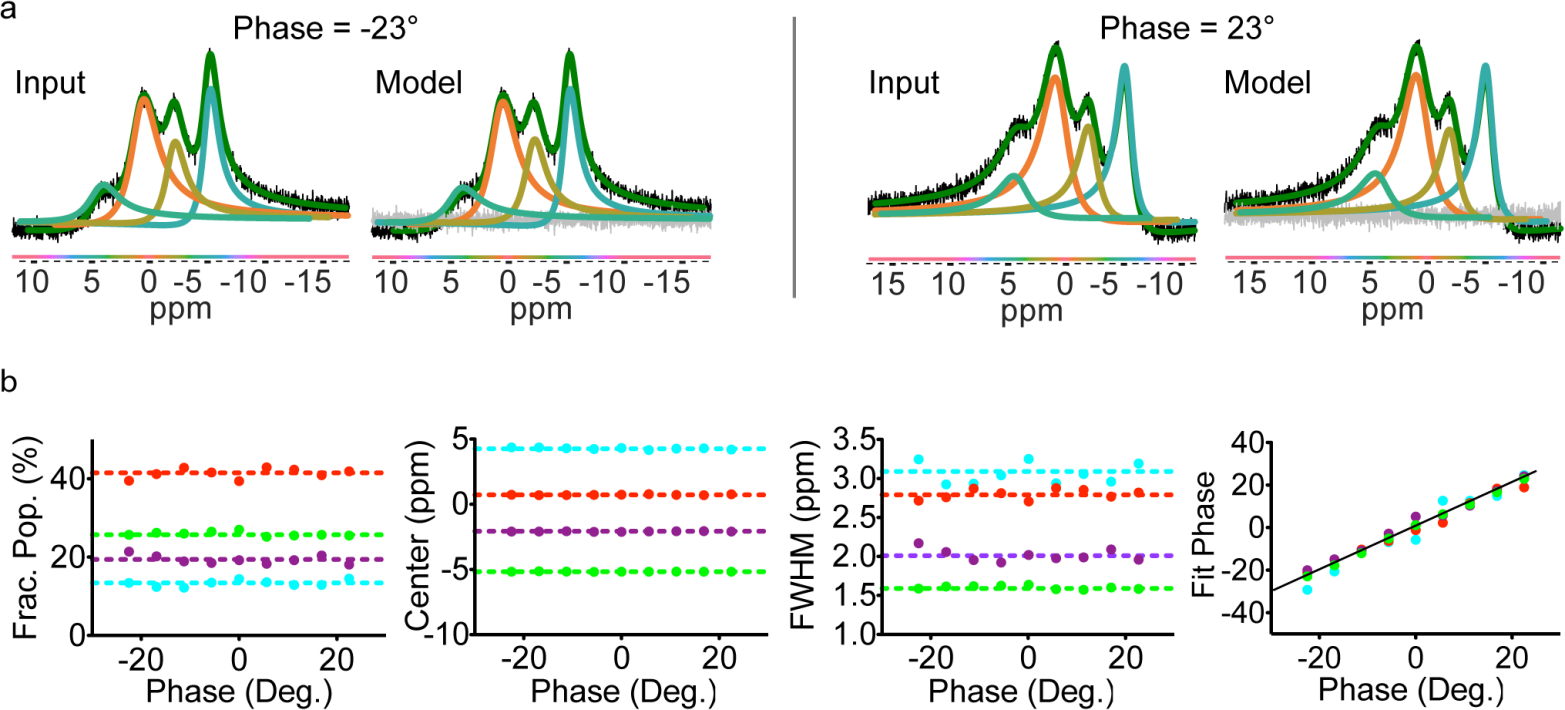
Out-of-phase data are well fit by *decon1d*. Simulated data (input) were fit with *decon1d* allowing the phase to vary (model). a) The input and fit models are nearly identical for these incorrectly phased simulated spectra. b) The fractional population, center, full width at half maximum peak height (FWHM) and phase of the simulated spectrum (dashed and solid lines) and the fits (colored dots) were graphed as a function of the phase of the simulated spectrum (x-axis). In general these fits are not adversely affected by poor phasing. The difference between the data and the fit (residual error) is shown in grey and the sum of individual fitted peaks is shown in green.

Incorrect phasing can be hard to discern in broad, low signal-to-noise 1D NMR spectra. Therefore, we tested the impact that incorrect phasing has on our fitting method. Four simulated NMR spectra each with nine zero order phases between π/8 and –π/8 radians were generated using an in house MATLAB script (see Methods) and fit using *decon1d*. In general, fits of these improperly phased simulated spectra were very similar to fits of properly phased spectra (Fig 4A). As above, both fixed and free phase model selection was carried out. The fixed phase models overestimated the number of peaks, splitting the four actual peaks into many peaks for all but the in-phase spectrum (not shown). Similarly, deconvolution utilizing iNMR also produced overfitting even in slightly out of phase peaks (misphased by π/32 radians, S7 Fig). As might be expected, deconvolutions carried out with the additional parameter of variable phase produced lower BIC scores than fixed phase models on the out of phase spectra. Modeled peak phases clustered tightly around the true phase, indicating that *decon1d* may be used to determine the correct phasing of experimental spectra (Fig 4B). This could be accomplished via deconvolution of the spectrum at several relative phases. The fit phase values could be used to interpolate the zero phase value for the spectrum. Thus model selection using *decon1d* is robust for even imperfectly phased spectra.

### Impact of chemical exchange on accuracy of fit

The fitting method in *decon1d* assumes pure Lorentzian lineshapes. Exchange between two magnetic environments, defined as p and p*, with chemical shift difference Δν near the rate of exchange between the two environments (k_ex_) leads to intermediate exchange lineshapes [4]. However, it has been shown that all transitions in all exchange regimes result in Lorentzian line shaped spectra that sum to give the overall observed lineshape. In the intermediate exchange regime, the Lorentzian peaks that make up the overall spectrum can have distinct relative phases and variable widths, chemical shifts, and heights [11]. In addition, for a single spin in the weak-coupling limit, each spin transition, or spectral peak, corresponds to a signal from the set of nuclei in a distinct chemical shift environment [11]. Using the LineShapeKin program [4], we produced simulated spectra of a single nuclei exchanging between two to four chemical shift environments (e.g. p and p*; p, p* and p**; etc.) with varying lifetime in each state (e.g. τ_p_ and τ_p*_) resulting in different equilibrium populations in each environment and overall exchange rate (e.g. k_ex_ = 1/τ_p_ + 1/τ_p*_) [12].

**Fig 5:**
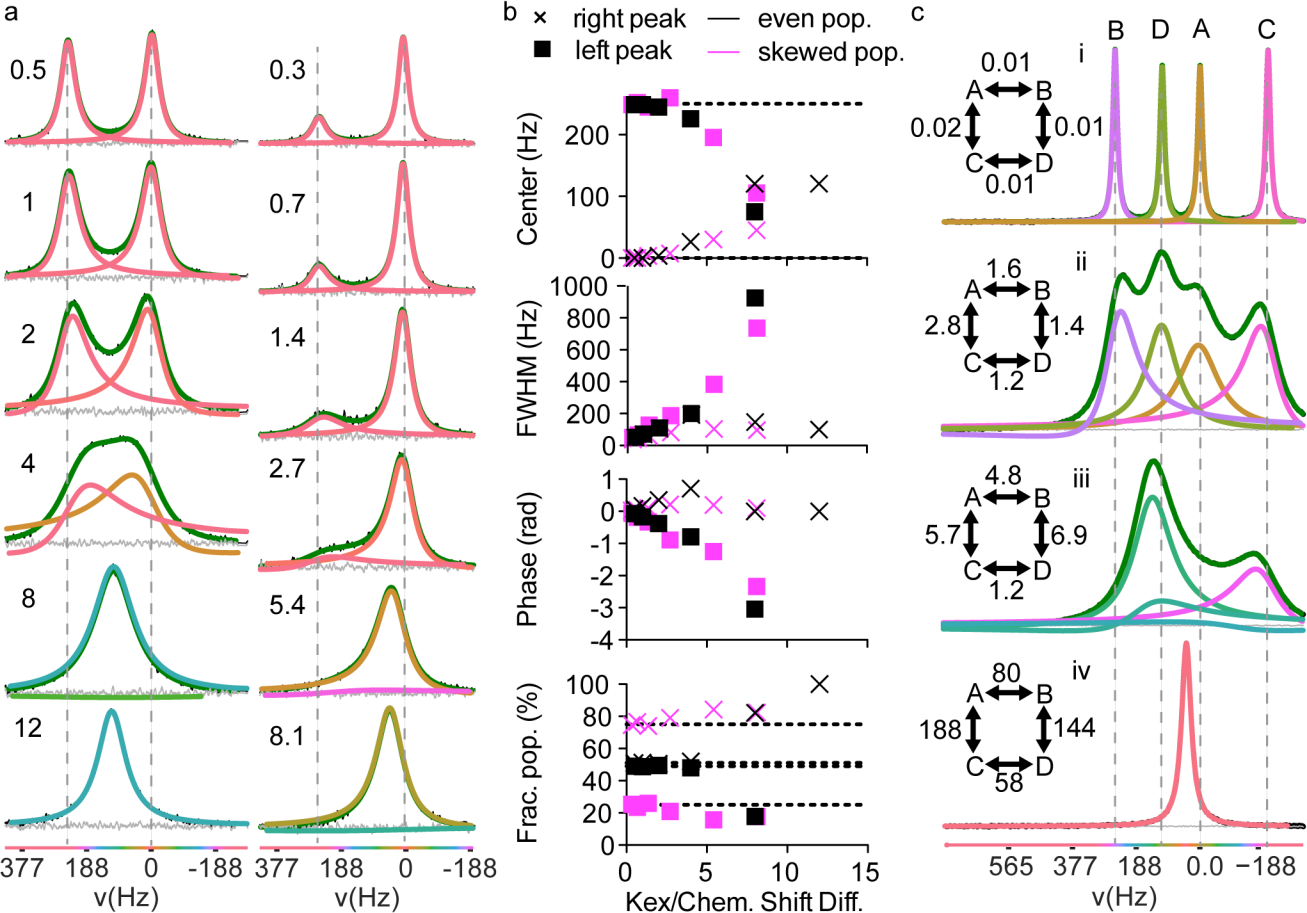
Intermediate exchange data are well fit by *decon1d*. Data were simulated using LineShapeKin and fit using *decon1d*. a) The spectrum from a single nucleus exchanging between two chemical shift environments was simulated with near equal populations (left panel; 48%:52%) and skewed populations (right panel; 25%:75%) at varying exchange rate to chemical shift difference values (k_ex_/Δδ, displayed numbers) and the best model of the component spectral lines was determined by *decon1d*. Vertical gray dashed lines indicate the true chemical shifts in the absence of exchange. b) Fitted parameters from the models in panel a. c) Simulated spectra from a single nucleus exchanging between four chemical shift environments with similar populations at varying k_ex_/Δδ values (displayed numbers) and the best model as determined by *decon1d*. The difference between the data and the fit (residual error) is shown in grey and the sum of individual fitted peaks is shown in green.

In the case of two exchanging populations, the best model found by *decon1d* predicted either one or two populations (Fig 5). As the ratio of exchange rate to the chemical shift difference between the environments (k_ex_/Δδ) is increased from near zero (slow exchange) to ~4 (just before coalescence of the peaks), both the chemical shifts and the relative populations are largely preserved. However, as k_ex_/Δδ is increased beyond 4, the lineshape increasingly takes on fast exchange characteristics and both the chemical shift and relative population information is progressively lost as the two signals merge to form one Lorentzian peak (Fig 5). The relative phase of the two peaks in the fitted model becomes increasingly divergent as k_ex_/Δδ is increased and upon nearing 8 the phases differ by ~π radians. In addition the out of phase peak becomes very broad. This outcome was consistent for both evenly split and skewed p and p* populations. Thus it appears that *decon1d* predictions containing relatively narrow, oppositely phased peaks (e.g. Fig 2C) are likely unphysical. In practice, when a model consisting of relatively sharp inverted peaks is the result, another fit should be attempted with the phase limited to a smaller range (e.g. < ±π/4). In future versions of *decon1d*, we will incorporate methods to link limits on phase and peak width so that severely out-of-phase peaks are only modeled if they have larger widths relative to other, in-phase peaks. In contrast to the results using variable phase models, most fixed phase models (i.e. with phase error limited to < ±π/50) incorrectly predicted more than two peaks for simulated spectra with strong intermediate exchange effects (S8 Fig).

We also tested deconvolution of more complex spectra involving exchange of one nucleus between four chemical shift environments (Fig 5C, not shown). Deconvolution of these spectra produced similar results to those for two-site exchange (Fig 5A); all fits contained fewer than four component peaks. As fast exchange is approached, information on the chemical shift and fractional populations found at each chemical shift environment are progressively lost from the spectra. However, in cases where k_ex_/Δδ is smaller than ~8, deconvolution of the spectrum can indicate the presence of more than one population. In summary, line-broadened NMR spectra due to chemical exchange are well fit by *decon1d* when the phase is free to vary, including in heavily skewed populations, and can be expected to find a reasonable lower bound for the number of chemical shift environments sampled by a nucleus regardless of the exchange kinetics.

Information extracted from a spectrum via *decon1d* could be used as a starting point for simulation of candidate kinetic schemes. Other currently available programs are capable of simulation and in some cases fitting user-defined kinetic schemes of intermediate exchange data with Matlab add-ons, such as LineShapeKin [4] and NMRkin [13], or the stand alone program MEXICO [14]. These programs are especially useful for kinetic analysis from series data where the relative populations of component states are systematically varied (e.g. through step-wise addition of a ligand to progressively shift the protein population from free to bound). In contrast, the *decon1d* program reported here aims to extract as much information as possible about the relative populations in each magnetic environment (i.e. thermodynamics) from a single spectrum without imputation of a user-specified kinetic scheme.

### Use of *decon1d* to probe a protein’s conformational ensemble

**Fig 6:**
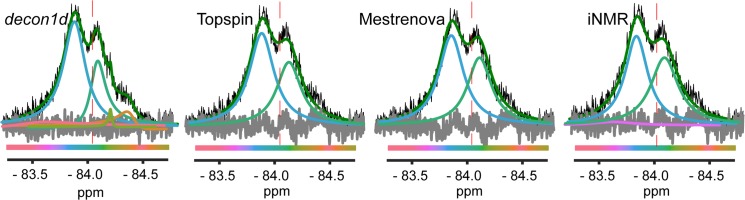
Fit of real experimental data. The ligand binding domain of PPARγ C285S/K474C was treated with BTFA and then NMR was performed at 298K. Deconvolution of the ^19^F NMR signal was carried out using the indicated programs. In each case the difference between the data and the fit (residual error) is shown in grey and the sum of individual fitted peaks is shown in green.

We recently reported 2D and 3D heteronuclear NMR studies of the apo-, or ligand-free, ligand binding domain (LBD) of PPARγ—a ligand-responsive transcription factor and the target of FDA-approved insulin sensitizing drugs [15]. These studies revealed that an important region for protein-protein interaction, which includes the C-terminal “helix 12”, is dynamic on the intermediate exchange time scale [16, 17]. Thus peaks corresponding to residues in helix 12 were not visible, suggesting that this region of PPARγ samples at least two conformations in the absence of a ligand. To probe the conformations accessed by helix 12, we attached 3-bromo-1,1,1-trifluoroacetone (BTFA) to a PPARγ LBD double mutant (C285S, K474C), where a native cysteine was removed and a new cysteine was introduced in helix 12 (PPARγ-BTFA). A ^19^F NMR signal of PPARγ-BTFA is detectable, but very broad with a total width of ~170 Hz at the half maximum of the signal (FWHM). Fitting two separate NMR spectra (acquired at ~376 MHz) with two separate protein samples using *decon1d* yields a prediction of five or six peaks, with 90±8% of the total area attributable to two main signals; these peaks have FWHM values of 89±2 Hz (61±3% of area) and 65±4 Hz (29±5% of total area) and are separated by 0.175±0.035 ppm (66±13 Hz) with slightly opposing phase (Fig 6, S9 Fig). In two-site exchange signal coalescence is expected when k_ex_/Δδ ~ 4.44 [18]. In this case with resolved peaks separated by 66±13 Hz, coalescence would be expected at a k_ex_ (= k_-1_+k_1_) of 290±58 s^-1^. Therefore these data suggest that PPARγ-BTFA helix 12 is switching between two long-lived conformations at an exchange rate slower than ~250 s^-1^ and that in addition minor populations of PPARγ exist with helix 12 in at least two other minor conformations It should be noted that the intrinsic linewidth of a non-exchanging fluorine label attached to PPARγ could be on the order of tens of hertz. This non-exchange linewidth adds to the uncertainty in chemical shift differences between the two peaks and thus the exchange rate. Two other minor conformations (total of 5–8% of the total signal fractional population) of 50±21 Hz and 11±1 Hz FWHM are predicted at very consistent chemical shifts (-84.40±0.01 ppm and 84.21±0.00 ppm). A wide small peak (Fig 6) was predicted for only one of the repeated experimental datasets. We also fit these spectra using various commercially available programs and found that *decon1d* produced the lowest residual value and was unique in consistently predicting a narrower linewidth for the right peak centered at -84.1ppm (Fig 6, S9 Fig).

**Fig 7:**
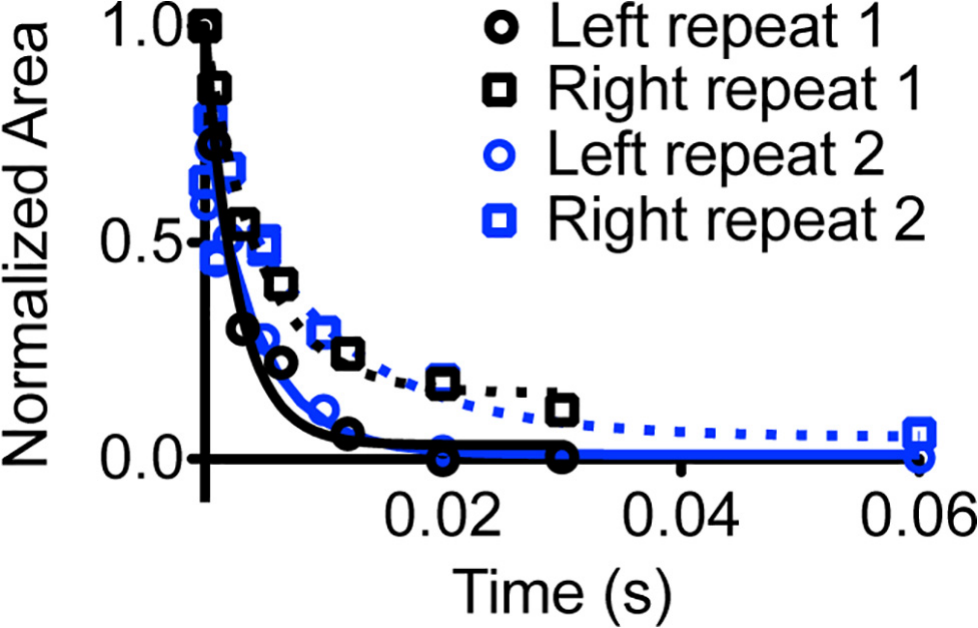
Fit of T_2_ relaxation data. The integral of the ^19^F NMR signal was calculated to the left of the dotted red vertical lines in Fig 6 (from -83 ppm to -84.05 ppm, left, circles) and to the right of the dotted red vertical lines in Fig 6 (from -84.05 to -85 ppm, right, boxes) in two independent experiments (blue and black) and plotted as a function of total delay time in the ^19^F T_2_ experiment described in the text. Two near-zero data points from repeat two at 0.3s delay are not shown for clarity (-0.012 and 0.023 for left and right respectively) but are included in the fits. Spectra used in the ^19^F T_2_ calculation are shown in supporting information (S10 Fig).

We next measured ^19^F transverse relaxation lifetime (T_2_) using a π/2 pulse followed by a variable delay, a π pulse, the same variable delay and finally ^19^F acquisition with ^1^H decoupling (S10 Fig) and compared these data to model predicted peak widths. We calculated T_2_ for two separate areas of the ^19^F signal envelope by integrating the signal area from -83.0 ppm to -84.05 ppm and separately for -84.05 ppm to -85.0 ppm (left and right of the red dashed line in Fig 6) and fit each area to a single exponential decay. Fitting of these data yielded T_2_ values that are distinct for each area (Fig 7) and predict a narrower peak for the right area, in closest agreement with both the predicted population-weighted arithmetic mean of all peaks to the right of -84.05 (62±2 Hz) and the linewidth of the large right peak (-84.08 ppm) in the decon1d model (61 Hz; Table 1). We also found that one of the programs, iNMR, produces two distinct fits depending on whether the “smooth” button is activated, one of which does predict peaks of various linewidth for the right area of the signal (S11 Fig). However the population-weighted arithmetic mean linewidth of peaks with centers in the right area of the signal envelope of the iNMR generated model (105 Hz) is inconsistent with the T_2_ data for the right portion of the signal envelope (95% CI 20–65 Hz). In addition deconvolution using both Topspin (Bruker) and Mestrenova software yields models that predict essentially equal relaxation on the left and right sides of the spectrum (Table 1). Thus, in this case without pre-specification of a specific kinetic model the *decon1d* method produced a model more consistent with experiment than other available fitting programs.

**Table 1:** Comparison of experimental to fit parameters.

	Fraction of total area	Center (ppm)	Width (Hz)
**LEFT PEAK**			
Experiment (T_2_)			90 (95% CI = 52–127)
*decon1d*	0.59	-83.88	90
Topspin	0.59	-83.87	99
MestReNova	0.57	-83.86	99
iNMR	0.50	-83.87	87
**RIGHT PEAK**			
Experiment (T_2_)			42 (95% CI = 20–65)
*decon1d*	0.25	-84.08	61
Topspin	0.41	-84.11	98
MestReNova	0.43	-84.11	98
iNMR	0.46	-84.11	105

The data from the two experiments depicted in Fig 7 were combined for this analysis.

In this analysis of these experimental spectra we have ignored the effects of any possible ^1^H-^19^F coupling on the spectra. Coupling was assumed to have negligible effect because protons were decoupled during ^19^F acquisition and the spectra appeared very similar with or without decoupling (not shown). This lack of coupling effect is probably due to small coupling constants compared to relatively wide ^19^F peaks. The effects of coupling may affect interpretation of the spectral deconvolution in other non-decoupled spectra with relatively strong coupling as compared to peak widths where multiple component peaks may represent a single structure [19].

## Conclusions, future developments, and availability

Although ^19^F labeling of biomolecules can greatly simplify NMR data of complex biological systems, even these more “simplistic” data can give rise to overlapping NMR signals that are difficult to deconvolute. In addition, the methods currently used for deconvolution of biological 1D ^19^F NMR spectra require significant user input and judgment. We have developed an objective method that uses statistically determined model selection to fit complex 1D NMR spectra packaged in the form of a Python-based program, *decon1d*. There are several areas that can be expanded in future developments of this approach. For example, we are currently exploring the application of constraints that can be placed on the phase of individual peaks depending on the their width and location relative to other peaks in order to avoid unphysical models. In addition, future work could incorporate auto-phasing of noisy, low signal-to-noise data. Finally, we have made this program publically available for the entire NMR community, and updates to this program will be made available from the project website (https://github.com/hughests/decon1d/).

## Methods

### Text file input preparation

It is important that the input data to the program is unmanipulated, non-line broadened data because the method assumes that the noise is normally distributed; the noise in line-broadened data does not typically follow a Gaussian distribution. The *decon1d* program was originally designed to use Bruker Topspin 3 text output files, however any text file of similar format will work. The text file should consist of two parts; the header and the intensity data. The header should be 10 rows. The fourth row should contain the left and right limit of the data (in ppm) as the fourth and eighth non-whitespace string or number from the left respectively. The sixth row should contain the total number of data points contained in the file as the fourth non-whitespace string or number from the left. In practice it may be easiest to just modify the header of the example text file included in the supplementary data, substituting in the left and right limits and the total number of data points. An example input file is given in supporting information (S1 Data). The second part of the text file contains the intensity information of the spectra in a column starting with the left limit intensity data at the top and ending with the right limit intensity data at the bottom. Inclusion of imaginary data is not necessary. To prepare this text file in Bruker Topspin 3, first display the region containing the signal of interest (with sufficient baseline data on both sides of the signal of interest to establish a baseline; see below). Next, right click on the displayed spectra and select “save display region to” from the menu, then select “a text file for use with other programs”. This will save a text file appropriate for use with *decon1d*.

### Additional information on the fitting procedure

#### Baseline correction:

The program subtracts the average intensity of the right and left ends of the spectrum (1/30^th^ of the total spectrum width at each end) from the entire spectrum and then adds the expected intensity (at the edge of the spectrum) of a spectrum centered single Lorentzian peak with height equal to the greatest intensity value in the spectrum and an estimated peak width based on the area between the spectrum and the x axis (the left Riemann sum).

#### Monte Carlo procedure:

The degree of perturbation to each parameter for each Monte Carlo refit is randomly chosen from a Gaussian distribution centered at the original value with variance proportional to the standard deviation of the original fit; i.e. with variance equal to the original standard deviation multiplied by a random number between 0 and 200 divided by the signal to noise ratio of the spectra.

### Simulated NMR data

Non-exchange broadened NMR data were simulated in a pseudo-random fashion using a MATLAB script where the number of peaks was either user defined or randomly selected from a uniform distribution on a user defined interval. Width, intensity, and center parameters for each peak were generated from user-defined normal distributions. An optional zero order phase offset, or set of phase offsets, was user-generated. For each phase offset, free induction decay (FID) vectors for each peak were generated using a sampling interval of 1 ms, an acquisition time of 1 s, and a ^19^F resonance frequency of 376.5 MHz. Also generated from this information were the corresponding ppm values. The FIDs for each individual peak within a phase offset were then summed. White Gaussian noise was imparted on the summed FID vector, which was subsequently Fourier transformed to afford the simulated NMR spectrum of a given zero order phase offset. The real part of the summed FID vector and the ppm vector were saved as a text file for input into *decon1d*. Peak parameters and zero order phase data were saved in separate text files for comparison to *decon1d* outputs. In addition, the intermediate exchange data were simulated using LineShapeKin [4] and MATLAB (www.mathworks.com) using the U (two chemical shifts) and U-R-RL (four chemical shifts) models. See supporting information (S1 Text) for details of simulation parameters.

### Experimental spectra acquisition and analysis

C285S K474C PPARγLBD was expressed in BL21(DE3) E. coli cells from a pET46-EK/LIC vector as a hexahistidine tagged protein. This construct consists of the LBD from residue 203 to the C-terminus (477; PPARγ isoform 1 residue numbering) with an N-terminus tag of MAHHHHHHVDDDDKMENLYFQG (histidine tag and TEV cleavage site). The protein was purified by Ni-NTA column and subsequently via a size exclusion column using buffer A (20mM KPO4, 50mM KCl, 0.5mM EDTA and 5mM TCEP) for elution. The ~100 μM protein was then treated with 80-fold molar excess of 3-bromo-1,1,1-trifluoroacetone (BTFA; ~8mM) at room temperature (22–25 C) for 3 h and then overnight at 4 C. Excess BTFA was subsequently removed by buffer exchange against buffer A, the protein was aliquoted and then frozen at -80°C. NMR was performed at 298K with acquisition at ~376 MHz on a Bruker room temperature BBFO probe with 10% D_2_O added. The spectrum displayed in Fig 6 and S9 Fig panel b was acquired for 13,000 scans with ~500uM protein. The spectrum displayed in S9 Fig panel a was acquired with 52,000 scans on ~250uM protein. Spectra were acquired with 10,000 and 12,000 scans for each delay in the two T_2_ experiments on ~500uM protein. The pulse sequence for acquisition of the T_2_ spectra is outlined in the text. The non-T_2_ experiment was similar, however the delay and π pulse were omitted. Over the course of the ~2.5 day acquisition of the T_2_ spectra some protein precipitation occurred resulting in a loss of ~20% of the signal. Identical short delay (20 μs) experiments were run at the beginning and after the end of the overall T_2_ experiment (S12 Fig) to quantify this loss and were used to adjust in a linear fashion the integral areas of the intervening variable delay experiments (which were run in randomized order). The correction assumed that precipitation occurred at a constant rate causing a linear decrease in signal over the ~2.5 day duration of each of the two T_2_ experiments. Area under the curve vs delay was plotted and fit using a single exponential decay fit in Prism (GraphPad Software Inc.). The deconvolution of the spectra was carried out on the spectrum from -82.0 to -85.5 ppm via specification of analysis limits within decon1d.

## Supporting Information

S1 Data Example data file for input into *decon1d*. This is the simulated spectrum data displayed in S4 Fig panel c. This simulated spectrum consists of five Lorentzian peaks with relative areas (percent of total area), centers (ppm) and widths (ppm) of (19.24, 3.961253, 0.6362), (21.0776, -5.064015, 0.4249), (18.31387, -5.503231, 0.8113), (18.63312, 3.048056, 0.6403) and (22.73541, 1.778537, 0.7756). All phases are equal to zero.
(TEXT)

S1 Fig Example of the input text format (see S1 Data for actual file). (TIF)

S2 Fig Initial test of *decon1d* and iNMR on simulated spectra. Fixed phase fit signifies limiting the phase to less than ±π/50 radians. Grey = residual and green = sum of individual peaks (expected to match data).
(TIF)

S3 Fig Initial test of *decon1d* and iNMR on simulated spectra. Fixed phase fit signifies limiting the phase to less than ±π/50 radians. Variable phase allows full freedom for phase. Grey = residual and green = sum of individual peaks (expected to match data). Alternate deconvolutions (with BIC values within 6 of those displayed) of the fixed phase model of column a and the variable phase model of column c are displayed in S5 Fig.
(TIF)

S4 Fig Initial test of *decon1d* and iNMR on simulated spectra. Fixed phase fit signifies limiting the phase to less than ±π/50 radians. Variable phase allows full freedom for phase. Grey = residual and green = sum of individual peaks.
(TIF)

S5 Fig Alternate models with a BIC score less than 6 greater than the model with the best BIC score. (TIF)

S6 Fig iNMR provides inconsistent fits for the same simulated data with varying signal to noise. f) Input simulated NMR spectra showing the true underlying peaks that make up the spectra. a-e) Fits utilizing iNMR of these same simulated data with varying signal-to-noise ratio: a) 5 b) 10, c) 25, d) 75 and e) 244. Signal-to-noise was calculated from the highest signal value divided by the root mean square value of the noise in a region devoid of signal.
(TIF)

S7 Fig Deconvolution of slightly misphased data leads to overfitting. iNMR was used to deconvolute the same simulated spectrum (composed of 4 peaks) as shown in Fig 4 (misphased by π/32 radians).
(TIF)

S8 Fig The fixed phase fit is inaccurate when the ratio of k_ex_ to chemical shift difference is between 1 and 8 (intermediate exchange). Deconvolution of the same simulated spectra as shown in Fig 5A was carried out with a) *decon1d* (phase error restricted to < ±π/50 radians) and b) with iNMR. Grey = residual and green = sum of individual peaks (expected to match data).
(TIF)

S9 Fig Alternate models of ^19^F PPARγC285S/K474C. a) Repeat of experiment shown in Fig 6. ^19^F NMR spectroscopy was carried out on a separate aliquot (from that used in the experiment displayed in Fig 6) of BTFA-treated PPARγ C285S/K474C and fitted using *decon1d*. Peak color is a visual aid for comparing peak location between graphs as it roughly indicates peak center in ppm as shown by the color bar. b) Alternate deconvolution of the spectrum displayed in Fig 6. The difference in BIC score between this model and the one displayed in Fig 6 is 5.99 indicating little support for this model.
(TIF)

S10 Fig Spectra from two separate experiments used in the *T*_*2*_ calculations. Numbers to the right indicate the length of the variable delay in milliseconds between a π/2 pulse and acquisition. Each experiment (of a given delay) was performed in randomized order.
(TIF)

S11 Fig Alternate deconvolution of real experimental data produced by iNMR when the smooth button is activated. (TIF)

S12 Fig Overlay of spectra (adjusted to same height) of the starting acquisition and an identical acquisition run after the end of the T2 experiments displayed in S10 Fig and analyzed in Fig 7. The integral of the area of the spectrum acquired immediately following the T_2_ experiment was 78% and 80% the area of the beginning spectrum of the T_2_ experiment, but was otherwise very similar for the two T_2_ experiments performed here. The signal decrease is due to protein precipitation over the course of the ~2.5 day experiments.
(TIF)

S1 Text Simulation parameters for intermediate exchange simulation using LineShapeKin. (PDF)
