# Supplementary figures and images for: Deconvolution of Complex 1D NMR Spectra Using Objective Model Selection
Source: PLoS One. 2015 Aug 4;10(8):e0134474. doi: 10.1371/journal.pone.0134474 (PMC4524620; doi:10.1371/journal.pone.0134474)

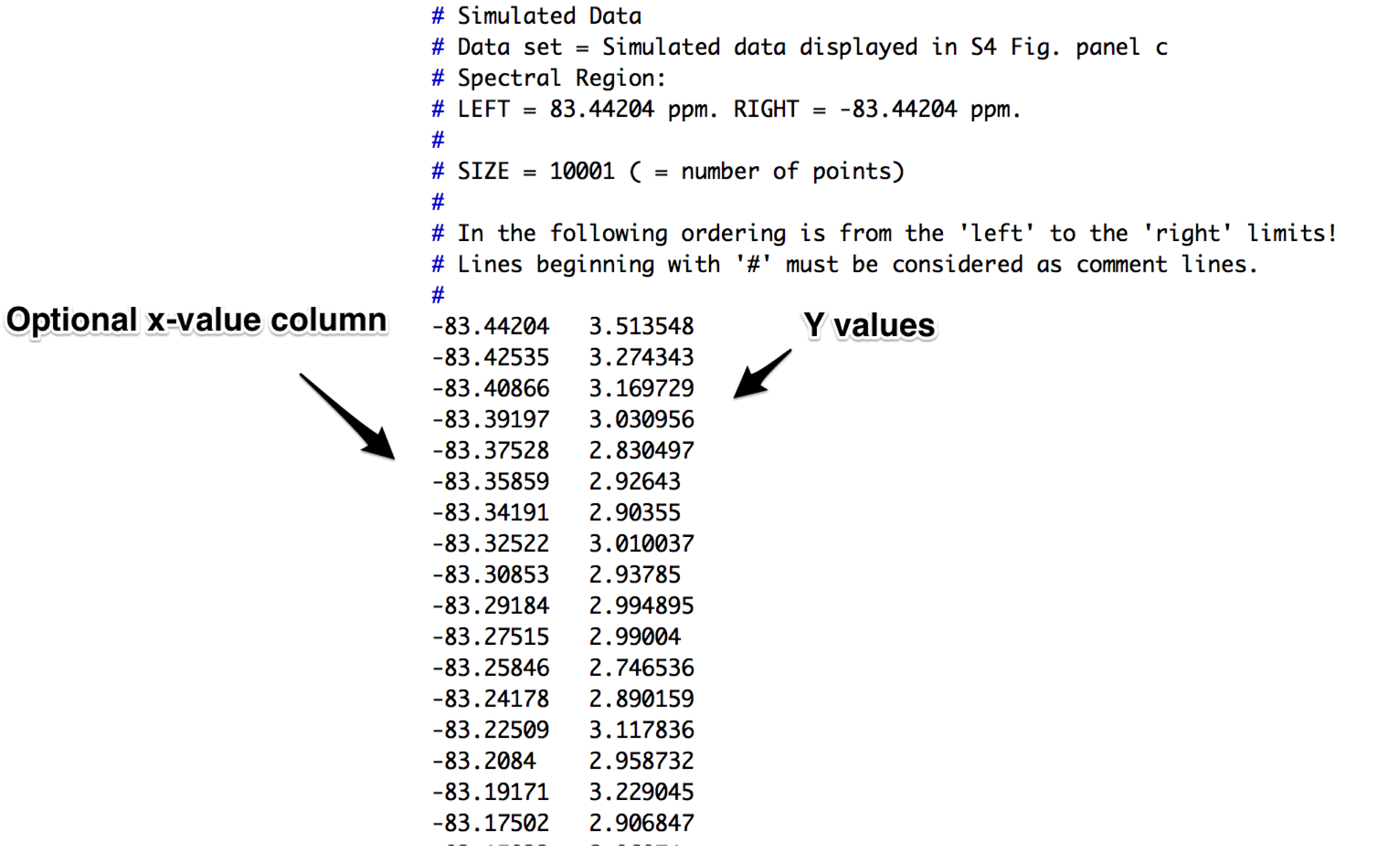

Supplement: S1 Fig — (TIF) [file pone.0134474.s002.tif]

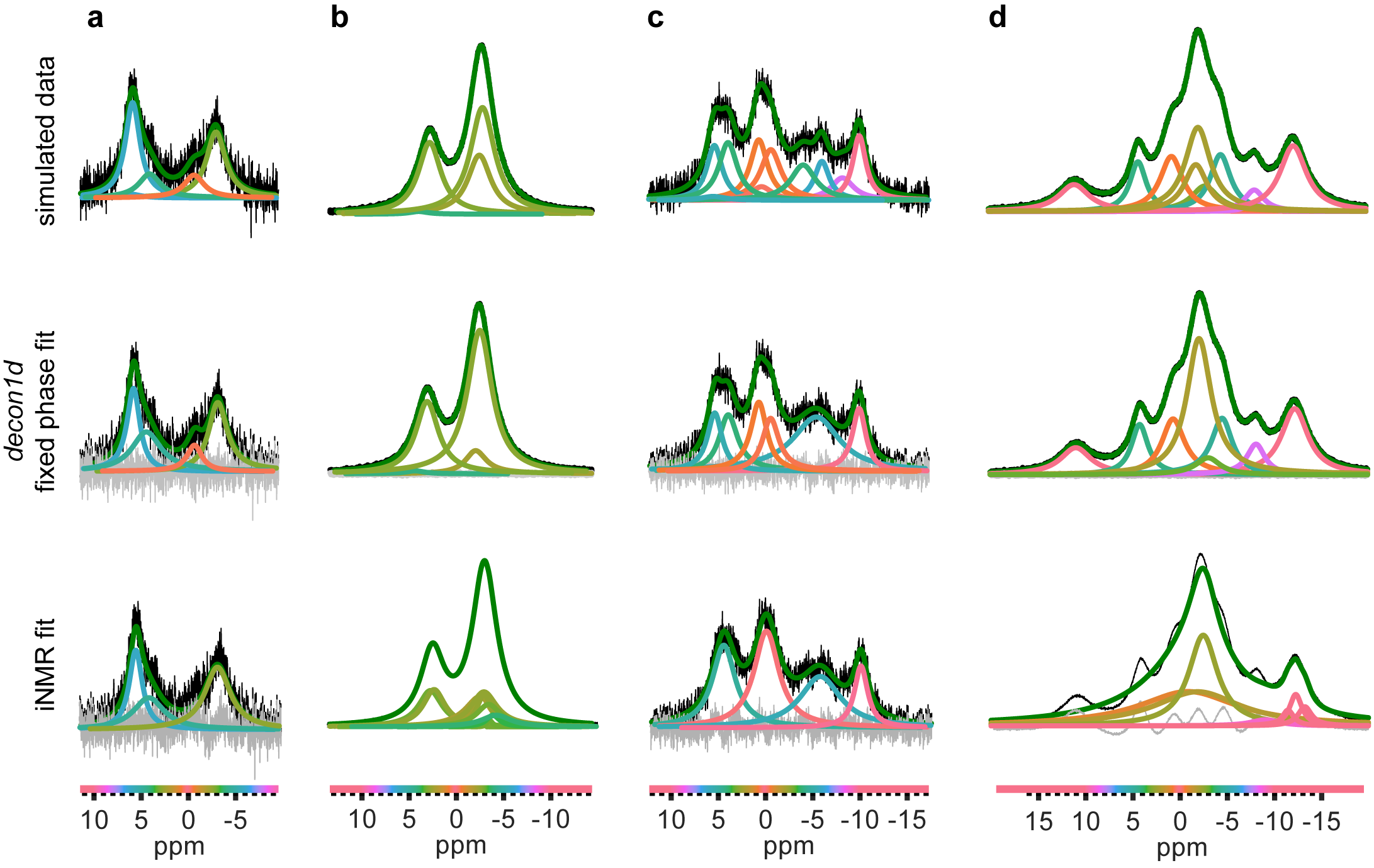

Supplement: S2 Fig — Fixed phase fit signifies limiting the phase to less than ±π/50 radians. Grey = residual and green = sum of individual peaks (expected to match data). (TIF) [file pone.0134474.s003.tif]

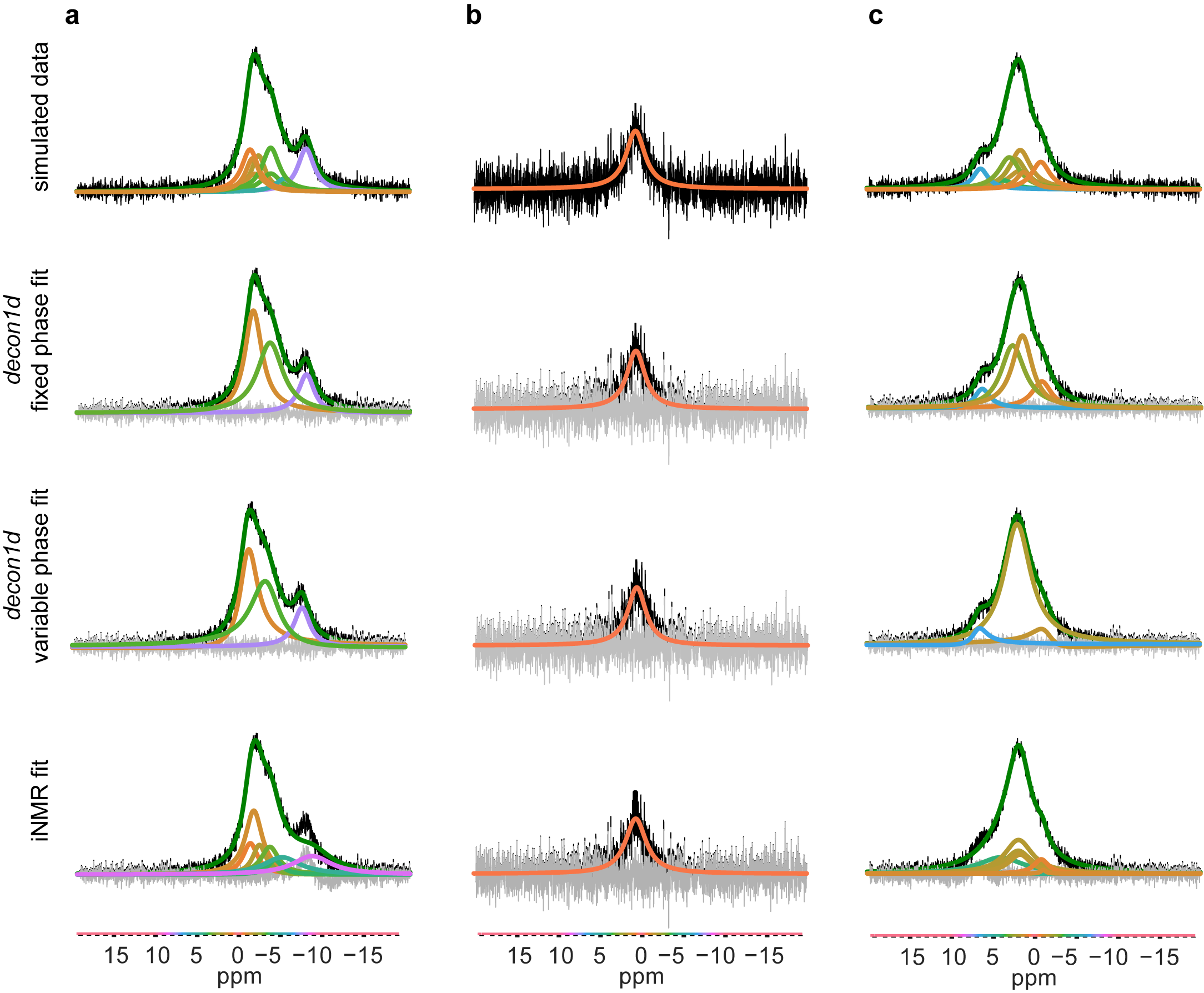

Supplement: S3 Fig — Fixed phase fit signifies limiting the phase to less than ±π/50 radians. Variable phase allows full freedom for phase. Grey = residual and green = sum of individual peaks (expected to match data). Alternate deconvolutions (with BIC values within 6 of those displayed) of the fixed phase model of column a and the variable phase model of column c are displayed in S5 Fig. (TIF) [file pone.0134474.s004.tif]

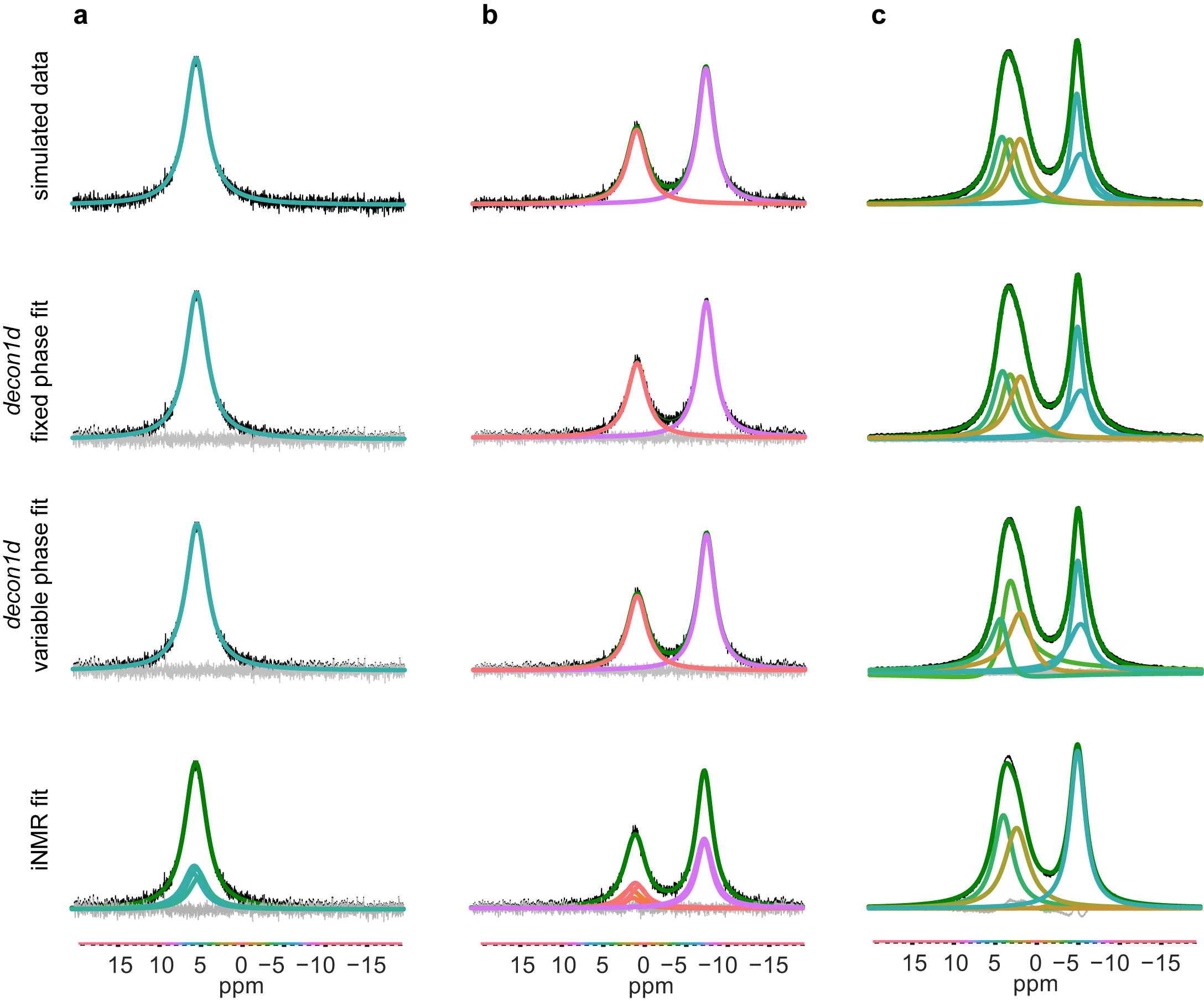

Supplement: S4 Fig — Fixed phase fit signifies limiting the phase to less than ±π/50 radians. Variable phase allows full freedom for phase. Grey = residual and green = sum of individual peaks. (TIF) [file pone.0134474.s005.tif]

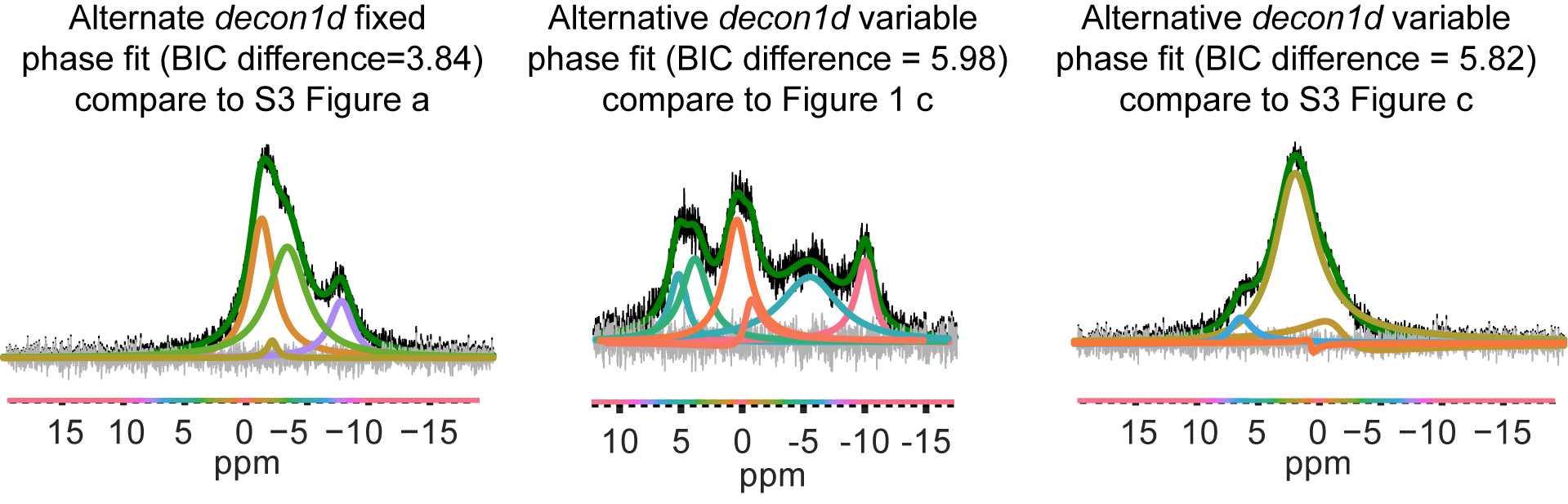

Supplement: S5 Fig — (TIF) [file pone.0134474.s006.tif]

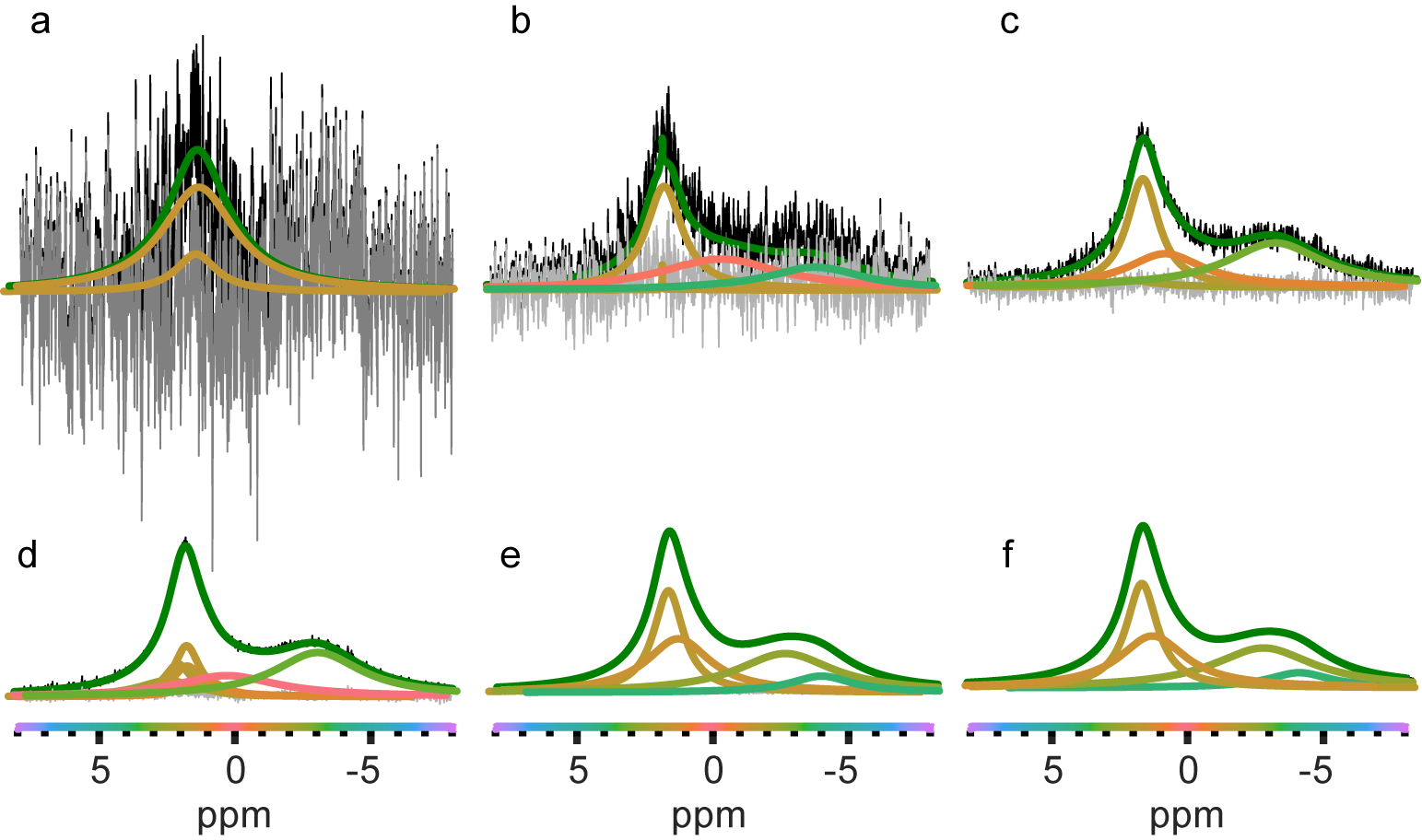

Supplement: S6 Fig — f) Input simulated NMR spectra showing the true underlying peaks that make up the spectra. a-e) Fits utilizing iNMR of these same simulated data with varying signal-to-noise ratio: a) 5 b) 10, c) 25, d) 75 and e) 244. Signal-to-noise was calculated from the highest signal value divided by the root mean square value of the noise in a region devoid of signal. (TIF) [file pone.0134474.s007.tif]

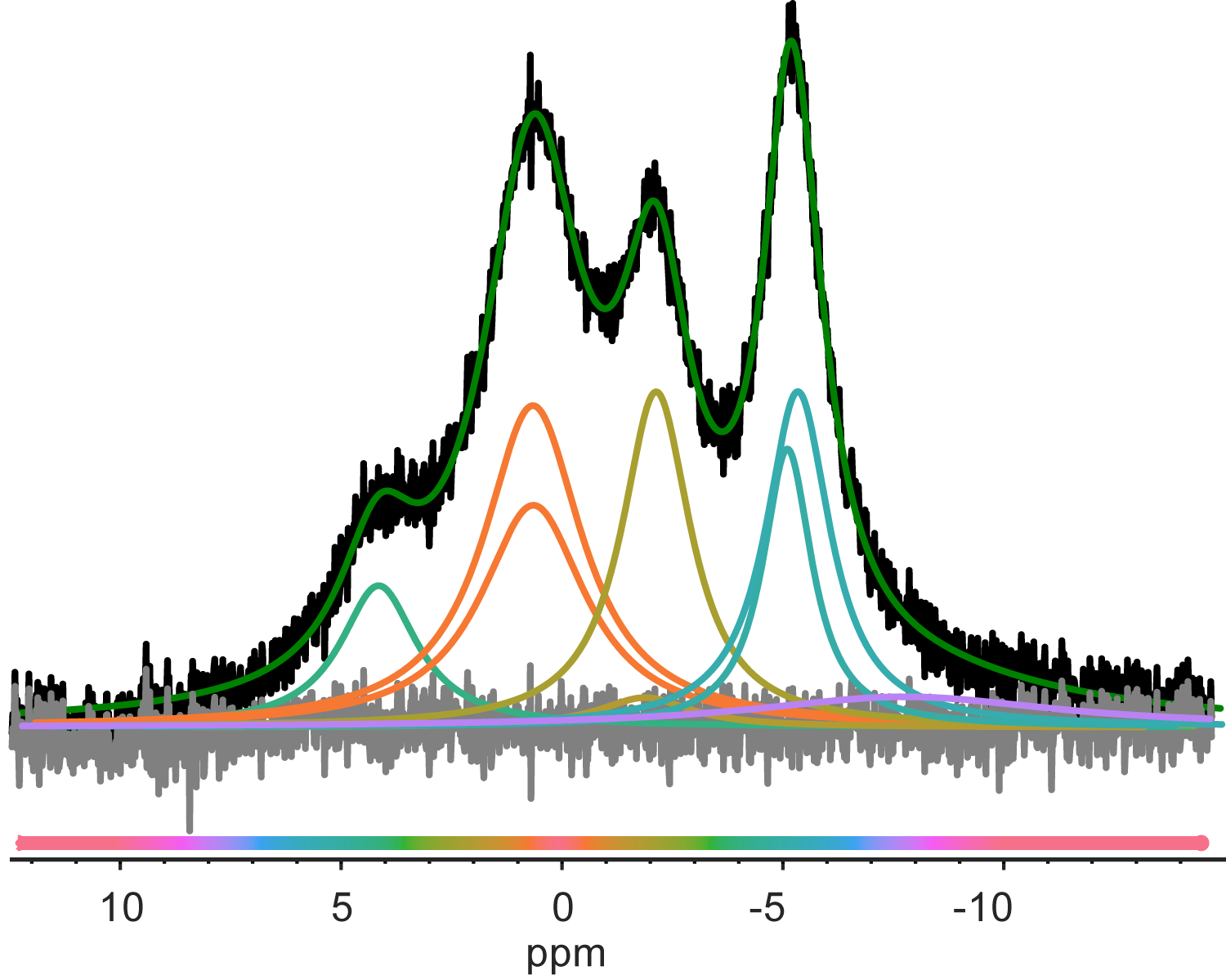

Supplement: S7 Fig — iNMR was used to deconvolute the same simulated spectrum (composed of 4 peaks) as shown in Fig 4 (misphased by π/32 radians). (TIF) [file pone.0134474.s008.tif]

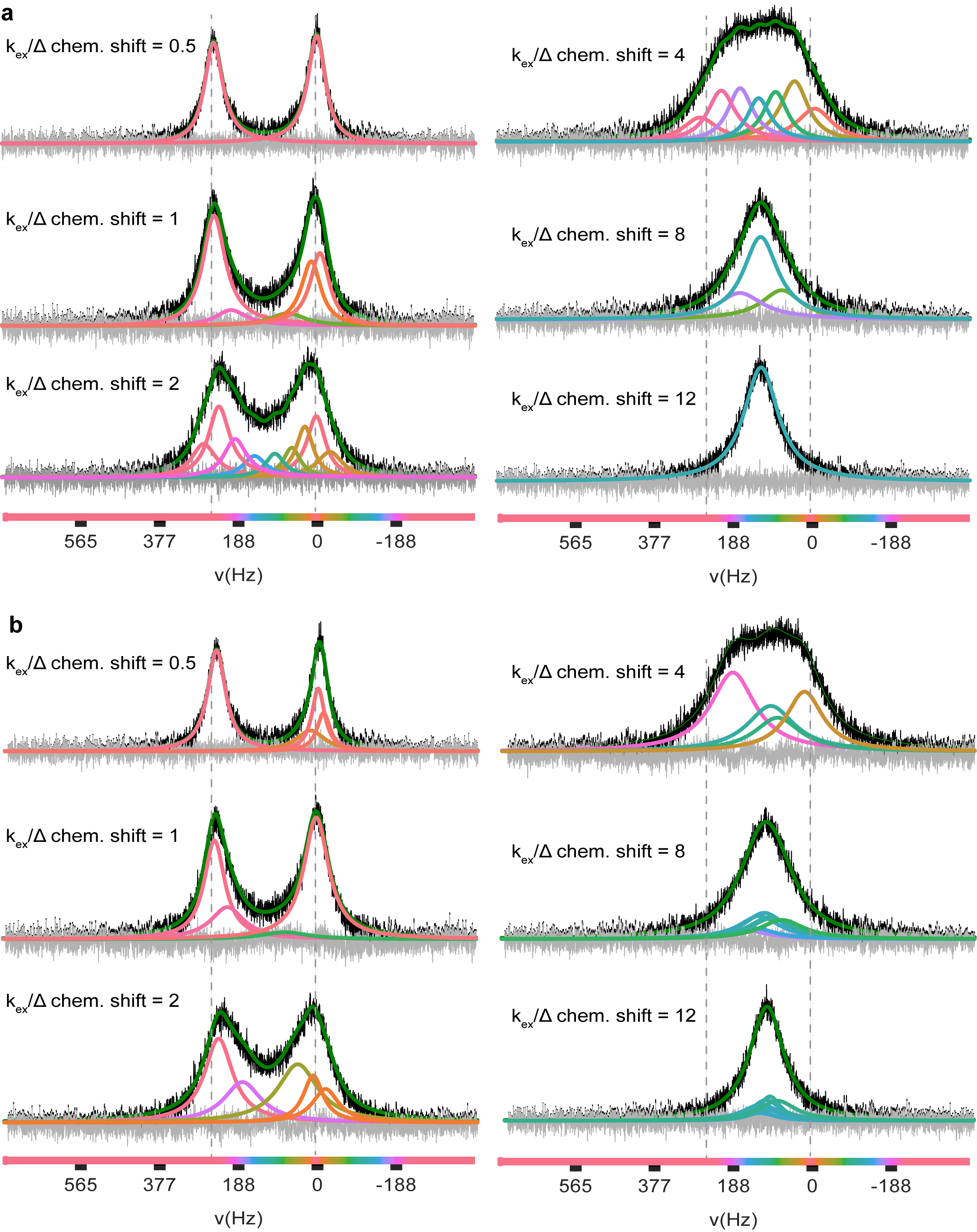

Supplement: S8 Fig — Deconvolution of the same simulated spectra as shown in Fig 5A was carried out with a) decon1d (phase error restricted to < ±π/50 radians) and b) with iNMR. Grey = residual and green = sum of individual peaks (expected to match data). (TIF) [file pone.0134474.s009.tif]

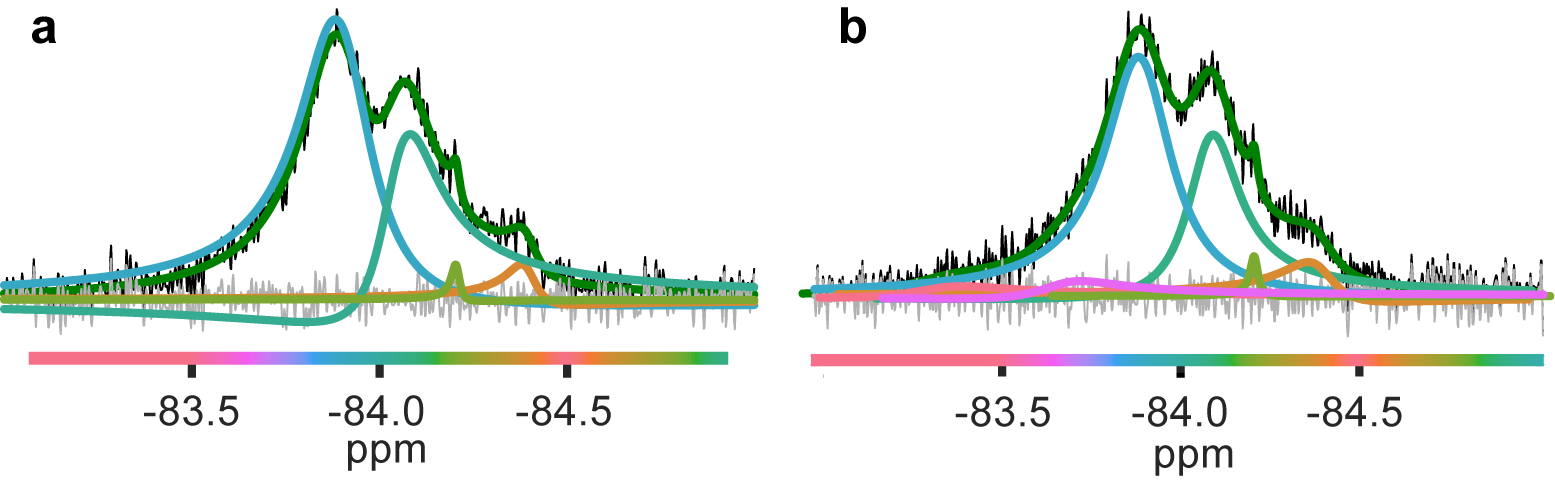

Supplement: S9 Fig — a) Repeat of experiment shown in Fig 6. 19F NMR spectroscopy was carried out on a separate aliquot (from that used in the experiment displayed in Fig 6) of BTFA-treated PPARγ C285S/K474C and fitted using decon1d. Peak color is a visual aid for comparing peak location between graphs as it roughly indicates peak center in ppm as shown by the color bar. b) Alternate deconvolution of the spectrum displayed in Fig 6. The difference in BIC score between this model and the one displayed in Fig 6 is 5.99 indicating little support for this model. (TIF) [file pone.0134474.s010.tif]

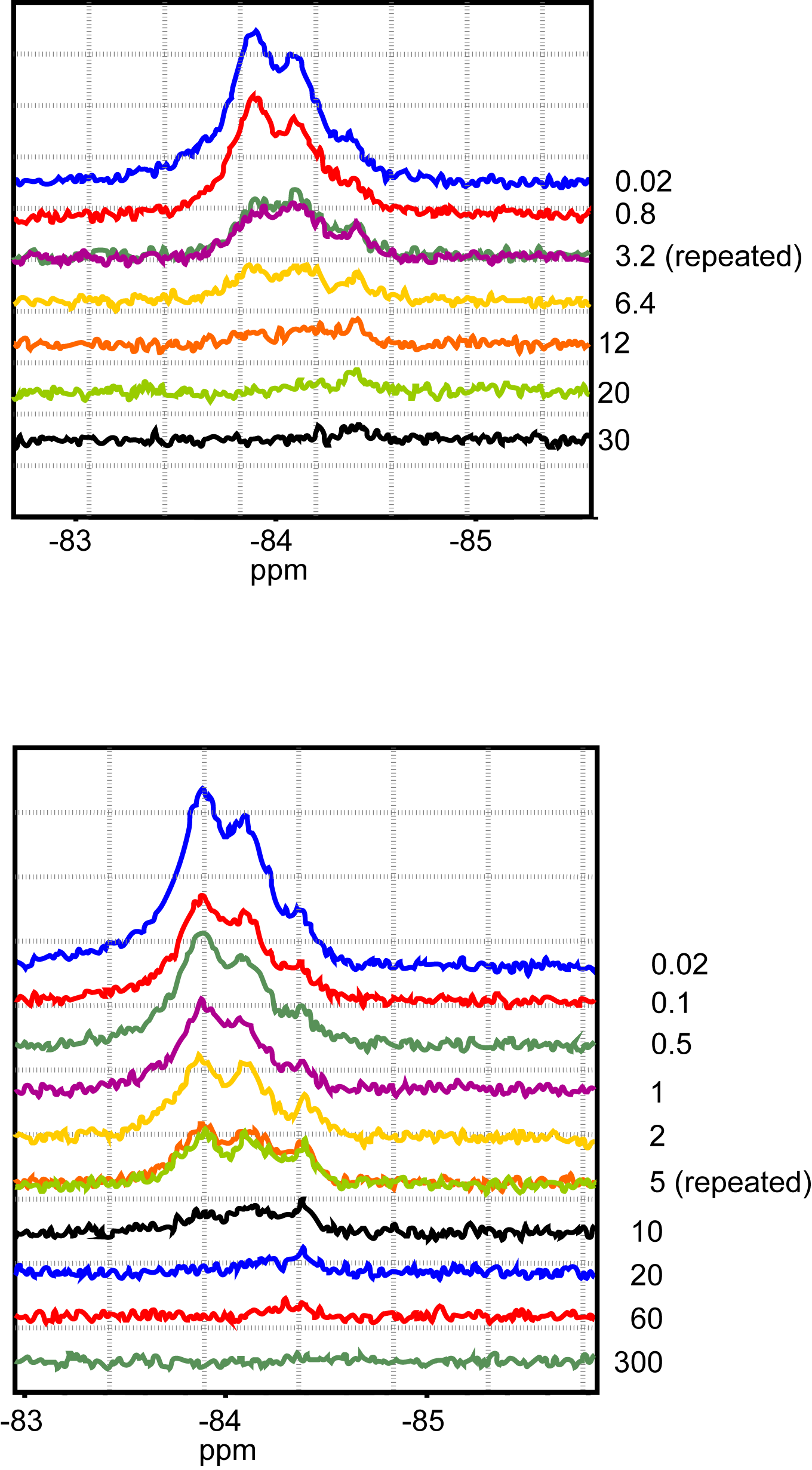

Supplement: S10 Fig — Numbers to the right indicate the length of the variable delay in milliseconds between a π/2 pulse and acquisition. Each experiment (of a given delay) was performed in randomized order. (TIF) [file pone.0134474.s011.tif]

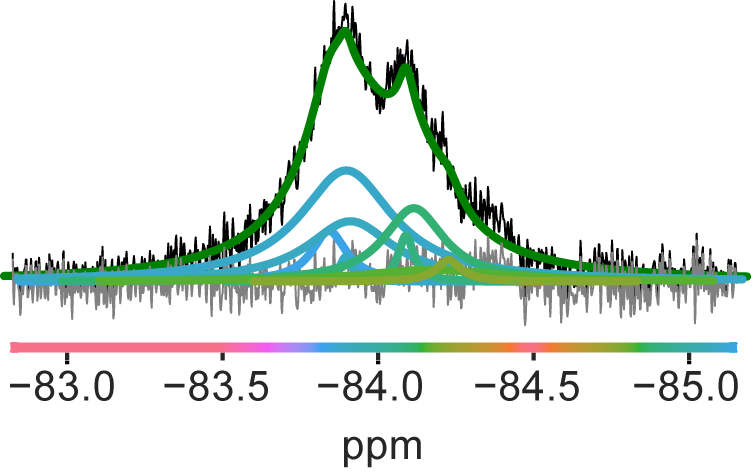

Supplement: S11 Fig — (TIF) [file pone.0134474.s012.tif]

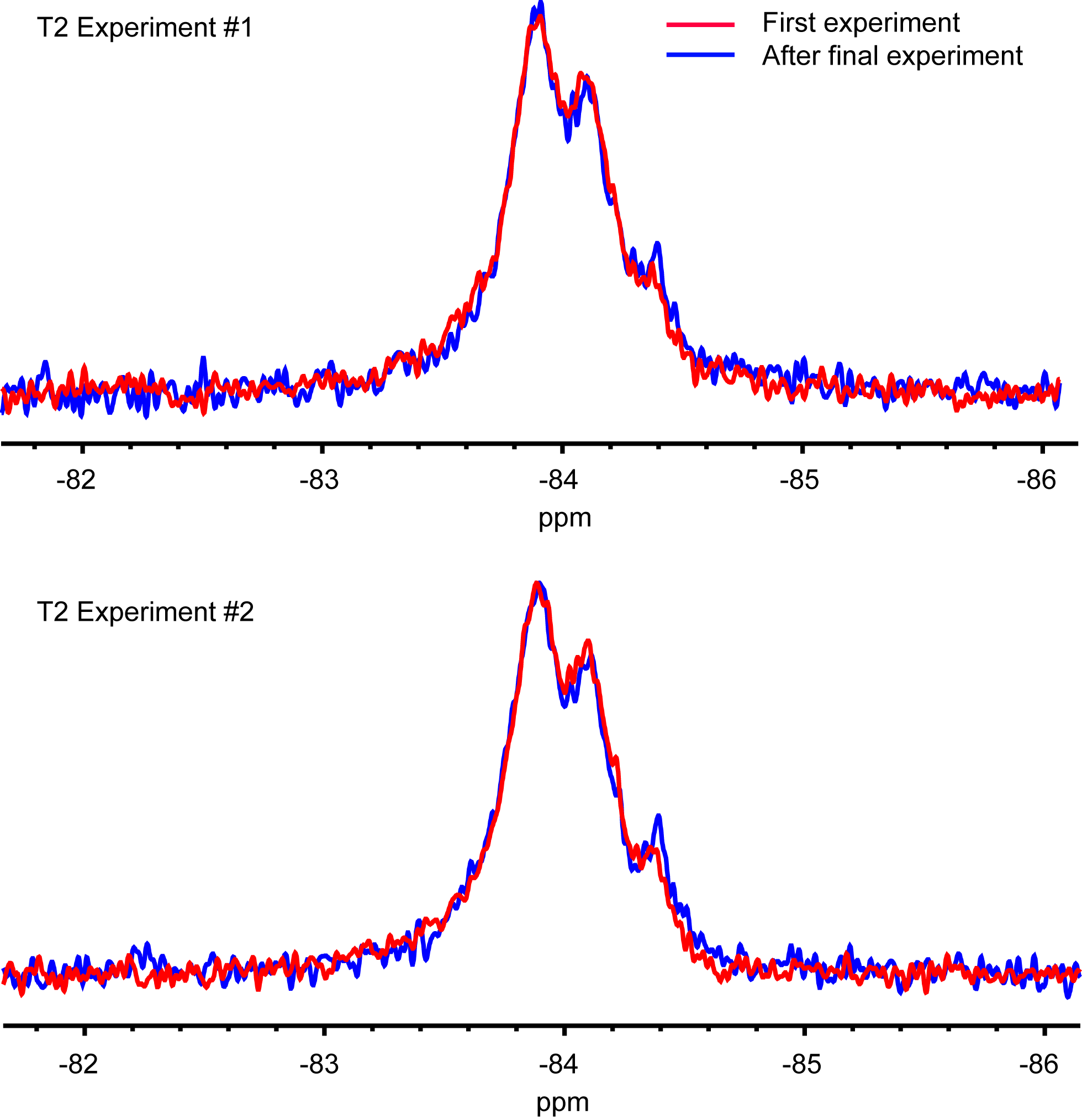

Supplement: S12 Fig — The integral of the area of the spectrum acquired immediately following the T2 experiment was 78% and 80% the area of the beginning spectrum of the T2 experiment, but was otherwise very similar for the two T2 experiments performed here. The signal decrease is due to protein precipitation over the course of the ~2.5 day experiments. (TIF) [file pone.0134474.s013.tif]
