# Supplementary material for: Deconvolution of Complex 1D NMR Spectra Using Objective Model Selection
Source: PLoS One. 2015 Aug 4;10(8):e0134474. doi: 10.1371/journal.pone.0134474 (PMC4524620; doi:10.1371/journal.pone.0134474)
Supplement: S1 Text — (PDF) [file pone.0134474.s014.pdf]

values highlighted in yellow are set in the setup file in LineShapeKin	Values highlighted in blue are set in the run file in LineShapeKin	Title: Deconvolution of complex 1D NMR spectra using automated model selection Journal: Travis S. Hughes1*, Henry D. Wilson2, Ian Mitchelle S. De Vera1 and Douglas J. Kojetin1<br>1 Department of Molecular Therapeutics, The Scripps Research Institute, Scripps Florida, Jupiter, Florida, 33458 USA. 2 Graduate Program at The Scripps Research Institute.																					
	Model	Chemical shift B (left) Rad/s	Chemical shift D Rad/s	Chemical Shift A Rad/s	Chemical shift C (right) Rad/s	Base relaxation rate for all (rad/s)	Spectral Window left (rad/s)	Spectral window right (rad/s)	Chemical shift B (left) Hz	Chemical shift D Hz	Chemical Shift A Hz	Chemical shift C (right) Hz	Base relaxation rate for all Hz	Spectral Window left (Hz)	Spectral window right (Hz)	Krev (per sec)	Kequ=Equilibrium constant (per molar)	Kfor (per sec molar) = cal from Krev and Kequ (Kfor=Kequ* Koff)	Free ligand [L] (calculated from total receptor, ligand and the Kequ)	Pseudo first order forward rate (Kfor*[L])	Total Receptor concentration (Molar)	Ratio of total ligand to total receptor	Number of simulated points
Figure 5a left panel (0.5)	U	1571			0	100	4730.9	-2365.5	250.032416			0	15.9154943	752.94612	-376.48102	63.8	1.00E+05	6.38E+06	9.6224E-06	61.4	1.00E-03	0.5	1.00E+04
Figure 5a left panel (1)	U	1571			0	100	4730.9	-2365.5	250.032416			0	15.9154943	752.94612	-376.48102	128	1.00E+05	1.28E+07	9.6224E-06	123.2	1.00E-03	0.5	1.00E+04
Figure 5a left panel (2)	U	1571			0	100	4730.9	-2365.5	250.032416			0	15.9154943	752.94612	-376.48102	255	1.00E+05	2.55E+07	9.6224E-06	245.4	1.00E-03	0.5	1.00E+04
Figure 5a left panel (4)	U	1571			0	100	4730.9	-2365.5	250.032416			0	15.9154943	752.94612	-376.48102	510	1.00E+05	5.10E+07	9.6224E-06	490.7	1.00E-03	0.5	1.00E+04
Figure 5a left panel (8)	U	1571			0	100	4730.9	-2365.5	250.032416			0	15.9154943	752.94612	-376.48102	1020	1.00E+05	1.02E+08	9.6224E-06	981.5	1.00E-03	0.5	1.00E+04
Figure 5a left panel (12)	U	1571			0	100	4730.9	-2365.5	250.032416			0	15.9154943	752.94612	-376.48102	1530	1.00E+05	1.53E+08	9.6224E-06	1472.2	1.00E-03	0.5	1.00E+04
Figure 5a right panel (0.3)	U	1571			0	100	4730.9	-2365.5	250.032416			0	15.9154943	752.94612	-376.48102	63.8	1.00E+05	6.38E+06	3.28E-06	20.9264	1.00E-03	0.25	1.00E+04
Figure 5a right panel (0.7)	U	1571			0	100	4730.9	-2365.5	250.032416			0	15.9154943	752.94612	-376.48102	128	1.00E+05	1.28E+07	3.28E-06	41.984	1.00E-03	0.25	1.00E+04
Figure 5a right panel (1.4)	U	1571			0	100	4730.9	-2365.5	250.032416			0	15.9154943	752.94612	-376.48102	255	1.00E+05	2.55E+07	3.28E-06	83.64	1.00E-03	0.25	1.00E+04
Figure 5a right panel (2.7)	U	1571			0	100	4730.9	-2365.5	250.032416			0	15.9154943	752.94612	-376.48102	510	1.00E+05	5.10E+07	3.28E-06	167.28	1.00E-03	0.25	1.00E+04
Figure 5a right panel (5.4)	U	1571			0	100	4730.9	-2365.5	250.032416			0	15.9154943	752.94612	-376.48102	1020	1.00E+05	1.02E+08	3.28E-06	334.56	1.00E-03	0.25	1.00E+04
Figure 5a right panel (8.1)	U	1571			0	100	4730.9	-2365.5	250.032416			0	15.9154943	752.94612	-376.48102	1530	1.00E+05	1.53E+08	3.28E-06	501.84	1.00E-03	0.25	1.00E+04
Figure 5c i	U_R_RL	1571	700	0	-1257	50	9461.8029	-4730.90145	250.032416	111.40846	0	-200.05776	7.95774715	1505.8927	-752.94635	See page 2	See page 2				1.00E-03	0.5	20000
Figure 5c ii	U_R_RL	1571	700	0	-1257	50	9461.8029	-4730.90145	250.032416	111.40846	0	-200.05776	7.95774715	1505.8927	-752.94635	See page 3	See page 3				1.00E-03	0.5	20000
Figure 5c iii	U_R_RL	1571	700	0	-1257	50	9461.8029	-4730.90145	250.032416	111.40846	0	-200.05776	7.95774715	1505.8927	-752.94635	See page 4	See page 4				1.00E-03	0.5	20000
Figure 5c iv	U_R_RL	1571	700	0	-1257	50	9461.8029	-4730.90145	250.032416	111.40846	0	-200.05776	7.95774715	1505.8927	-752.94635	See page 5	See page 5				1.00E-03	0.5	20000

```
#  Unique model identifier ### settings for Figure 5c iv
Model_code     U_R_RL

# Model description
Description  Case 2: Ligand bindig induces population shift

# Association constants
Ka_names   A1   A2   B1   B2
Ka         1e8  1.1  0.9  1.1

# Rate constants of REVERSE reactions
k_names     A1   A2     B1    B2
k2          10000    10000  10000   10000

# Names of NMR-active species
Species_names      R   R*   RL   R*L

# Names of NMR unobservable species
NMR_invisible_species_names     L

# Chemical shifts of pure species, 1/s
w0        0   -1257    1571  700

# Relaxation rates of pure species, 1/s
R2      50      50     50    50

# Heat of formation of the species, relative units
# The original species is a standard state with dH=0 !
dH     0    1    -2    -1
```

```
#  Unique model identifier  ### settings for Figure 5c iii
Model_code     U_R_RL

# Model description
Description  Case 2: Ligand bindig induces population shift

# Association constants
Ka_names   A1   A2   B1   B2
Ka         1e8  1.1  0.9  1.1

# Rate constants of REVERSE reactions
k_names     A1   A2     B1    B2
k2          600    200  300   500

# Names of NMR-active species
Species_names      R   R*   RL   R*L

# Names of NMR unobservable species
NMR_invisible_species_names     L

# Chemical shifts of pure species, 1/s
w0        0   -1257    1571  700

# Relaxation rates of pure species, 1/s
R2      50      50     50    50

# Heat of formation of the species, relative units
# The original species is a standard state with dH=0 !
dH     0    1    -2    -1
```

```
#  Unique model identifier  ### settings for Figure 5c ii
Model_code     U_R_RL

# Model description
Description  Case 2: Ligand bindig induces population shift

# Association constants
Ka_names   A1   A2   B1   B2
Ka         1e8  1.1  0.9  1.1

# Rate constants of REVERSE reactions
k_names     A1   A2     B1    B2
k2          200    200  150   100

# Names of NMR-active species
Species_names      R   R*   RL   R*L

# Names of NMR unobservable species
NMR_invisible_species_names     L

# Chemical shifts of pure species, 1/s
w0        0   -1257    1571  700

# Relaxation rates of pure species, 1/s
R2      50      50     50    50

# Heat of formation of the species, relative units
# The original species is a standard state with dH=0 !
dH     0    1    -2    -1
```

```
#  Unique model identifier   ### settings for Figure 5c i
Model_code     U_R_RL

# Model description
Description  Case 2: Ligand bindig induces population shift

# Association constants
Ka_names   A1   A2   B1   B2
Ka         1e8  1.1  0.9  1.1

# Rate constants of REVERSE reactions
k_names     A1   A2     B1    B2
k2          1    1  1   1

# Names of NMR-active species
Species_names      R   R*   RL   R*L

# Names of NMR unobservable species
NMR_invisible_species_names     L

# Chemical shifts of pure species, 1/s
w0        0   -1257    1571  700

# Relaxation rates of pure species, 1/s
R2      50      50     50    50

# Heat of formation of the species, relative units
# The original species is a standard state with dH=0 !
dH     0    1    -2    -1
```
